# Clinical and Epidemiological Profile of Herpes Zoster and Its Complications in a Tertiary Care Center of Bihar: A Prospective Study

**DOI:** 10.7759/cureus.43560

**Published:** 2023-08-16

**Authors:** Rajesh Sinha, Pinki Kumari, U.K. Pallavi, Subhasree Sarkar

**Affiliations:** 1 Dermatology, Indira Gandhi Institute of Medical Sciences, Patna, IND; 2 Ophthalmology, Indira Gandhi Institute of Medical Sciences, Patna, IND

**Keywords:** herpes zoster vaccine, herpes zoster oticus, herpes zoster ophthalmicus, zoster-associated pain, post-herpetic neuralgia, herpes zoster reactivation, varicella-zoster, herpes zoster virus

## Abstract

Background

Herpes zoster is a common viral infection caused by reactivation of the varicella-zoster virus (VZV) characterized by the presence of a segmental distribution of painful grouped vesicles on an erythematous base. It is associated with several complications like zoster-associated pain (ZAP), postherpetic neuralgia (PHN), pigmentary changes, scarring, secondary infections, and dermatosis as well as severe systemic complications.

Aims/objectives

The aim of the study was to analyze the various clinical and epidemiological patterns of herpes zoster and post-herpetic complications.

Materials and methods

We conducted a single-center observational cross-sectional study on 72 patients with herpes zoster and post-herpetic complications attending the dermatology outpatient department (OPD) to understand its various clinical and epidemiological patterns. A detailed history taking regarding the onset, progression, and complications of the disease, as well as the type, duration, and severity of pain, was taken, followed by a general physical, systemic, and cutaneous examination, along with investigations wherever needed.

Results

A total of 72 patients were included in the study, comprising 32 (44.4%) patients suffering from herpes zoster and 40 (55.5%) patients suffering from post-herpetic complaints. The minimum age was 14 years, the maximum age was 83 years, and the mean age in our study was 52 ± 17 years. The most commonly affected age group was 41-60 years. A total of 52 males and 22 females were included in the study, resulting in a male-to-female ratio of 2.3:1. The thoracic dermatome was the most commonly involved dermatome, observed in 43 (59.7%) patients, and the left side was more commonly affected, seen in 41 (56.9%) patients. Among the total 72 patients, 26 (36.1%) had co-morbidities, with hypertension (18%) being the most common, followed by diabetes mellitus (12.5%). Regarding the post-herpetic complaints encountered in our study, the most common was post-herpetic neuralgia, seen in 31 (77.5%) patients, followed by post-herpetic pigmentation (macular), observed in 22 (55%) patients, and scarring (papules, plaques, hypertrophic scar, and keloid), observed in 17 (42.5%) patients.

Conclusion

A broader understanding of the clinical and epidemiological factors of herpes zoster and post-herpetic complications is important as this disease constitutes a considerable burden in a tertiary care center and if not treated adequately, the after-effects might last for many years altogether. Hence, early diagnosis and initiation of adequate antiviral therapy as well as pain management is the key aspect of management.

## Introduction

Herpes zoster is a common viral infection caused by the reactivation of the varicella-zoster virus (VZV), which is known to remain dormant in the dorsal root ganglion following primary chickenpox infection or vaccination, and even intrauterine infection in rare cases [1]. The disease is characterized by the presence of a segmental distribution of painful grouped vesicles on an erythematous base along a specific dermatome [2]. While it was previously regarded as a condition affecting the elderly, its incidence among the younger population is also quite common [3]. The disease tends to be self-limiting in healthy individuals, but in extremely immunocompromised patients, it may manifest as multi-dermatomal, severe, vesiculo-pustular, nodular, crusted, ecthymatous, and widely disseminated, leading to chronic debilitation [4].

Various complications, including pain, postherpetic neuralgia (PHN), pigmentary changes, scarring in the form of hypertrophic or keloidal scars, secondary infections, as well as severe complications such as acute retinal necrosis, blindness, cerebellar ataxia, Guillain-Barre syndrome, herpes zoster ophthalmicus (HZO), Ramsay-Hunt syndrome, meningoencephalitis, stroke, and myocarditis, have been reported in the literature due to the involvement of ophthalmic, cerebral, or splanchnic nerves [5,6,7,8]. Our study aims to investigate the clinical and epidemiological patterns of herpes zoster and various post-herpetic complications.

## Materials and methods

Study type and period

A prospective cross-sectional study was conducted at a tertiary care hospital in Bihar during the period of May 2022 to June 2023 after ethical committee approval (Letter no: 584/IEC/IGIMS/2022).

Study design

All patients with herpes zoster and post-herpetic complications attending the dermatology outpatient department (OPD) were included in the study. Appropriate written informed consent was obtained in the vernacular language. Patient particulars as well as pre-existing co-morbidities were documented. This was followed by a detailed history-taking regarding the onset of the disease, primary symptoms and lesions, progression, and complications. The type of pain, duration of pain, and its severity were recorded using the Visual Analog Scale by Hayes and Patterson (1921) [9], where patients self-assessed their pain on a scale from zero to ten (ranging from no pain to unbearable pain). The severity of pain was categorized as mild for scores one to three, moderate for scores four to six, and severe for scores seven to ten. Subsequently, a general physical, systemic, and cutaneous examination was conducted. Necessary investigations such as Tzanck smear, blood panels, viral markers, and other relevant tests needed to confirm the diagnosis or establish any aggravating factors were performed as required.

Statistical analysis

The data were recorded and analyzed by using IBM SPSS Statistics for Windows, Version 26 (Released 2018; IBM Corp., Armonk, New York, United States) and Microsoft Excel (Microsoft Corporation, Redmond, Washington, United States). Tabulation, mean, standard deviation, and percentages were calculated.

## Results

**Figure 1:**
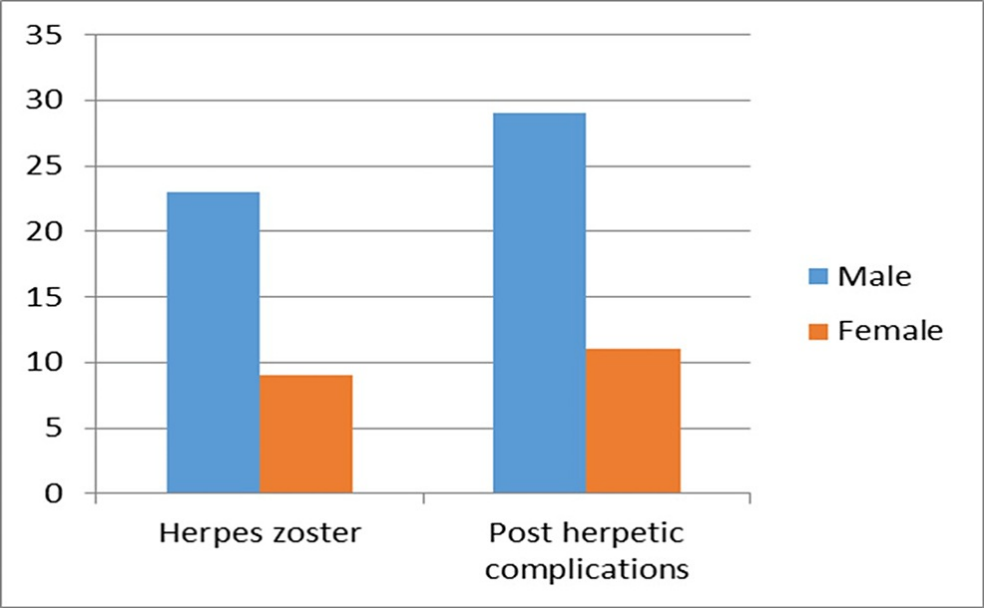
Number of patients with herpes zoster and post-herpetic complications

A total of 72 patients were included in the study, which comprised 32 (44.4%) patients with herpes zoster and 40 (55.5%) patients with post-herpetic complaints (Figure 1).

The minimum age was 14 years and the maximum age was 83 years and the mean age in our study was 52 ± 17 years. The most commonly affected age group was 41-60 years (Table 1). A total of 52 males and 22 females were included in the study and the male-to-female ratio was 2.3:1 (Figure 1).

**Table 1:** Age distribution

Age ( in years)	Number of patients and percentage (%)
<20	5 (6.9%)
20-40	10 (13.8%)
41-60	35 (48.6%)
61-80	18 (25%)
>80	4 (5.5%)

**Figure 2:**
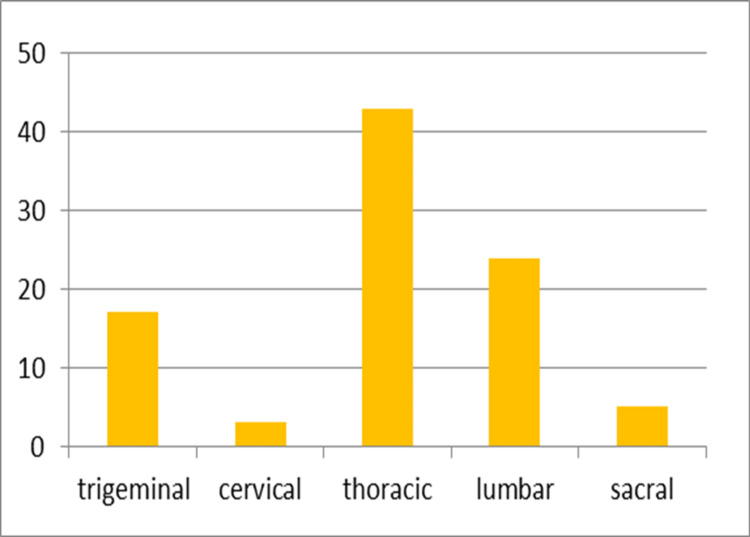
Frequency of involvement of various dermatomes

The maximum number of cases (43.7%) of herpes zoster was seen in the months between June and August. Thoracic dermatome was the most commonly involved dermatome and was observed in 43 (59.7%) patients, and the left side was more commonly involved and was observed in 41 (56.9%) patients (Figure 2).

**Figure 3:**
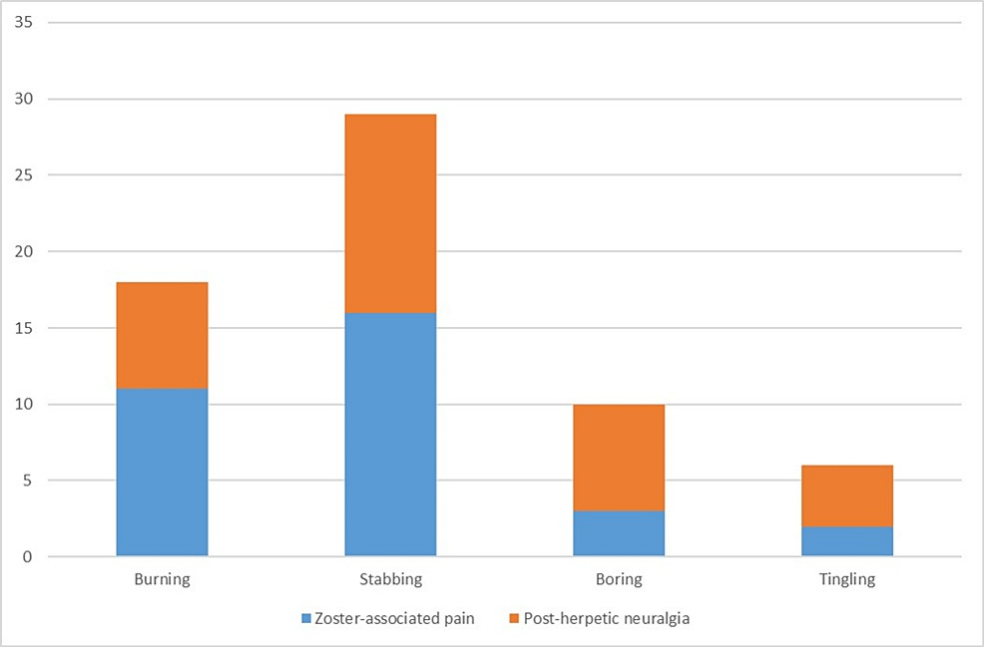
Type of zoster-associated pain and post-herpetic neuralgia reported

Multi-dermatomal involvement was observed in 23 (31.9%) patients (Table 2). The most frequently reported prodromal symptoms were mild fever and arthralgia, and pain was the initial presenting symptom before the appearance of a skin rash in 54 (75%) patients. Among those with acute herpes zoster rash, the most prevalent type of pain reported was stabbing, noted in 16 (50%) patients, followed by a burning sensation, described by 11 (34.3%) patients (Figure 3).

**Table 2:** Number of dermatomes involved

Number of dermatomes involved	Number of patients and percentage (%)
Uni-dermatomal	47 (65.2%)
Multi-dermatomal	24 (33.3%)

**Figure 4:**
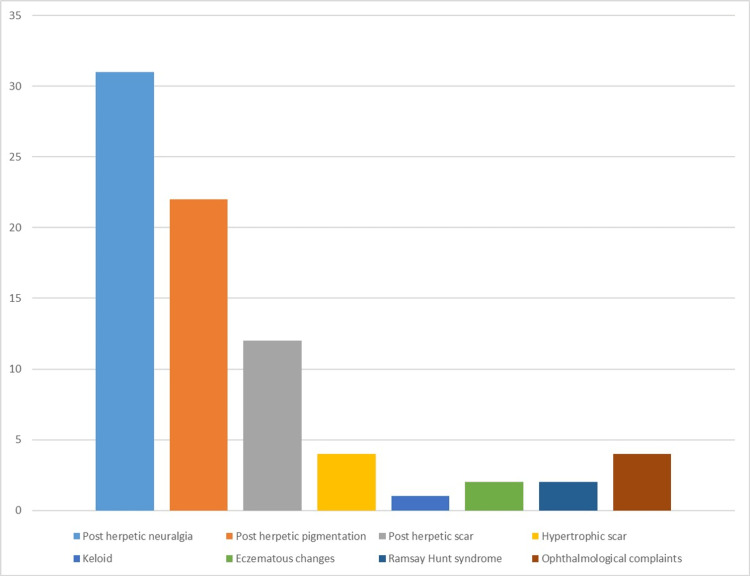
Types of post-herpetic complications observed

The pain was described as severe in intensity in 20 (62.5%) patients. Loss of sleep and hampering of day-to-day activities were reported in 22 (68.75%) patients. A history of previous chickenpox infection was present in 17 (23.6%) patients, and none of the patients had received a vaccine for varicella zoster or herpes zoster infection. Out of the total 72 patients, 26 (36.1%) had co-morbidities, with hypertension (18%) being the most common, followed by diabetes mellitus (12.5%). Two patients had a history of coronary artery disease, and two had chronic kidney disease. Two patients with known malignancies, one with papillary carcinoma of the thyroid and one with ovarian carcinoma, were observed (Table 3). In this study, all complications arising four weeks after the primary herpetic rash have been categorized as post-herpetic complications. Among the post-herpetic complaints encountered in our study, the most common was post-herpetic neuralgia, seen in 31 (77.5%) patients, followed by post-herpetic pigmentation (macular) in 22 (55%) patients and scarring (papules, plaques, hypertrophic scar, and keloid) observed in 17 (42.5%) patients (Figure 4). Most patients complained of both post-herpetic neuralgia and post-herpetic pigmentation or scarring (37.5%). The most common type of post-herpetic neuralgia reported was stabbing, noted in 13 (41.9%) patients, followed by a burning type in 7 patients (22.5%), and the intensity was most commonly described as moderate in 18 (58%) patients (Figure 3). The maximum duration of persistence of post-herpetic pigmentation and post-herpetic neuralgia observed in our study was seven years and three years, respectively.

**Table 3:** Frequency of various co-morbidities observed

Co-morbidities	Number of patients and percentage (%)
Hypertension	13 (18%)
Type 2 diabetes mellitus	9 (12.5%)
Coronary artery disease	2 (2.7%)
Bronchial asthma	1 (1.3%)
Chronic kidney disease	2 (2.7%)
Rheumatoid arthritis	1 (1.3%)
Hypothyroidism	1 (1.3%)
Papillary carcinoma of thyroid	1 (1.3%)
Benign prostatic hyperplasia	1 (1.3%)
Neuropathic pain	1 (1.3%)
Nephrotic syndrome	1 (1.3%)
Ca ovary with ascites	1 (1.3%)

Two patients presented with Ramsay-Hunt syndrome and one patient showed development of eczematous changes over the post-herpetic scar. Ophthalmological involvement was seen in four patients. On ocular examination, all four patients developed conjunctivitis. Two patients also developed anterior uveitis and keratitis.

## Discussion

Herpes zoster is caused by the reactivation of the dormant neurotrophic human alpha herpes virus known as varicella-zoster (VZV), also referred to as human herpes virus 3. Reactivation commonly occurs due to a decline in cell-mediated immunity associated with aging or due to immunosuppression caused by disease or medications [1]. The incidence of herpes zoster is age-dependent, ranging from 1.2 to 3.4 per 1000 persons per year among younger adults to 3.9-11.8 per 1000 persons per year in elderly patients (i.e., >65 years) [10]. While it was previously considered predominantly a disease of the elderly, its occurrence has become increasingly common among young individuals, as demonstrated in numerous prior studies [3,4,11,12]. This trend is also evident in our study, where patients ranged in age from 14 to 83 years. This shift has been attributed to mass vaccination efforts and the immunological status of individuals during the primary infection [13].

A total of 52 males and 22 females were included in the study, resulting in a male-to-female ratio of 2.3:1 (Figure 1). This could be attributed to a higher prevalence of health-seeking behavior and awareness among males compared to females in our region. The highest number of herpes zoster cases (43.7%) was observed between June and August, potentially due to the seasonal variation of herpes zoster associated with increased ultraviolet (UV) exposure.

**Figure 5:**
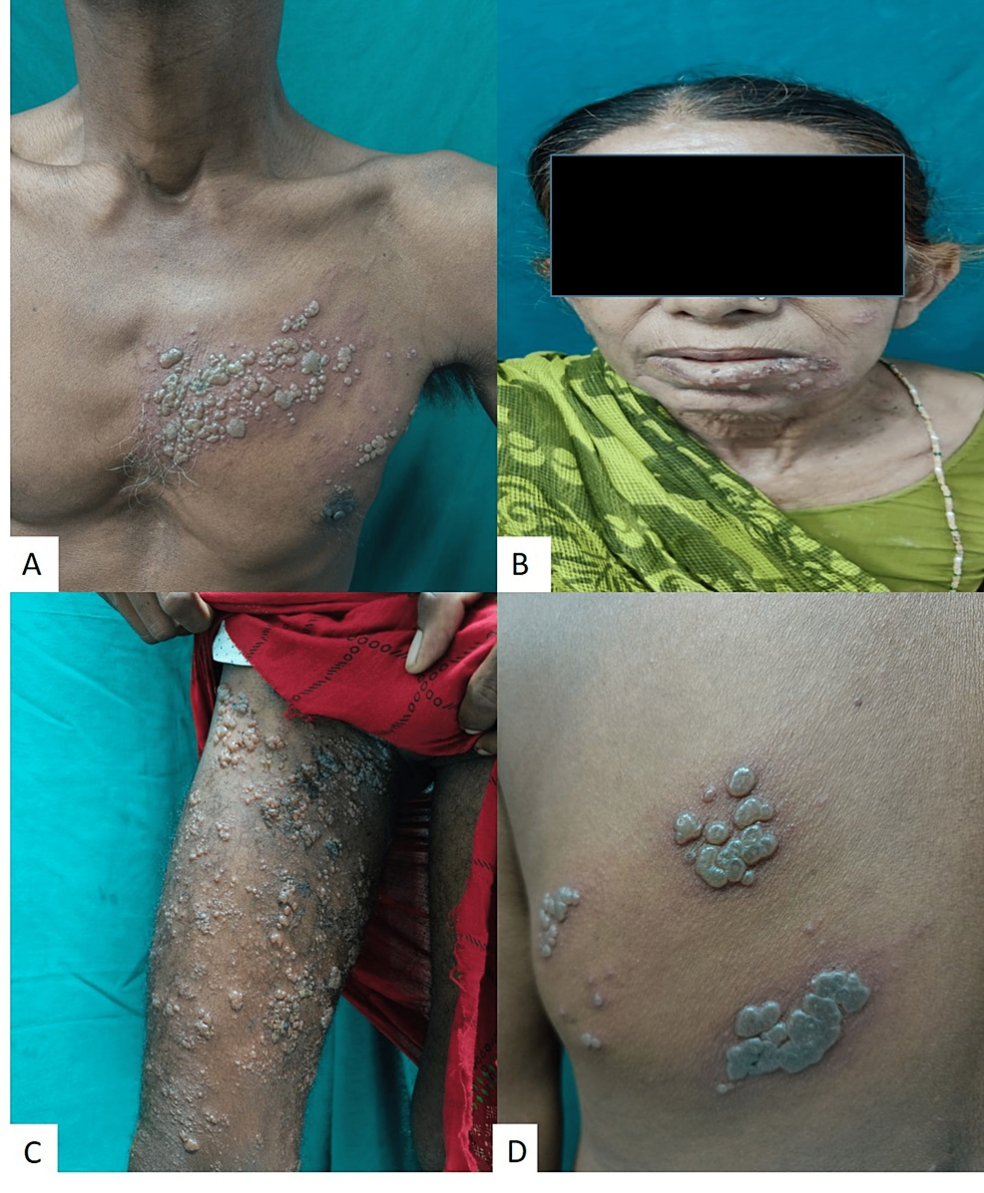
Clinical image showing various dermatomal involvement of herpes zoster A: thoracic, B: mandibular division of trigeminal nerve, C: lumbar, D: thoracic.

The disease characteristically presents as painful grouped vesicles on an erythematous base along a specific dermatomal distribution that does not cross the midline, giving rise to its name "zoster" or girdle, signifying its segmental arrangement [2]. In our study, the thoracic dermatome was the most frequently affected, observed in 43 (59.7%) patients, and the left side was more commonly involved, seen in 41 (56.9%) patients (Figure 2 and Figure 5). This finding was consistent with several previous studies [4, 12, 14, 15]. Following thoracic dermatome involvement, the lumbar dermatome was affected in 24 (33.3%) patients. Cranial nerve involvement was noted in 17 (23.6%) patients, with the maxillary division of the trigeminal nerve being the most commonly affected. Multi-dermatomal involvement was observed in 24 (33.3%) patients, among whom 37.5% were immunocompromised. The most common combination of affected segments was the thoracic and lumbar regions (Table 2).

The most commonly reported prodromal symptoms were mild fever and arthralgia. Pain is a highly prevalent characteristic in herpes zoster cases, often manifesting as deep, boring, pricking, electric shock-like sensations, burning, or shooting. It can also manifest as allodynia, and in certain instances, it may precede the appearance of skin lesions by several days to weeks, occasionally being the sole manifestation [5]. The conventional explanation for pain and allodynia involves cutaneous inflammation triggering nociceptive pain signals, alongside de-afferentation due to the necrosis of the infected dorsal root ganglion [16]. This pain can at times imitate conditions like angina, pleurisy, cholecystitis, appendicitis, duodenal ulcer, or renal colic, potentially leading to unnecessary interventions. A more contemporary term in use is zoster-associated pain (ZAP), which encompasses all pain occurring before or after the emergence of the herpes zoster rash [17].

Fifty-four (75%) patients recalled pain as their initial presenting symptom before the emergence of the skin rash. Among those with acute Herpes zoster rash, the most common type of pain described was a stabbing sensation, reported by 16 (50%) patients, followed by a burning-like sensation in 11 (34.3%) patients (Figure 3). The pain was noted as severe in intensity by 20 (62.5%) patients. A total of 22 (68.75%) patients reported experiencing sleep disturbances and hindrance of day-to-day activities due to the pain. The progression of the skin rash was characterized by the evolution from erythematous papules or macules to vesiculation, pustulation, and subsequent crusting. The severity of the rash was classified as severe in 9 (28.1%) patients and moderate in 14 (43.7%) patients. For the majority of patients (78.1%), the rash resolved within a span of two to three weeks. A history of previous chickenpox infection was reported by 17 (23.6%) patients, and none of the individuals had received a vaccine for varicella-zoster or herpes zoster infection.

**Figure 6:**
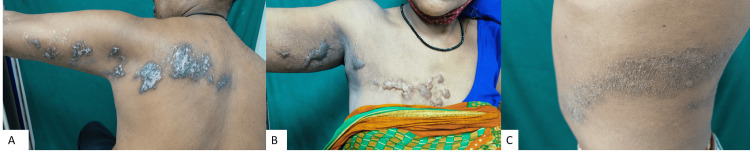
Clinical image showing various post-herpes dermatological complications A: scarring, B: keloid formation, C: eczematous change.

Several complications can arise from this infection, such as post-herpetic neuralgia (when pain persists for more than four weeks), along with aesthetically displeasing hypertrophic, keloidal, or atrophic scars, pigmentary changes, as well as secondary infections [6]. In this study, any complications emerging four weeks after the primary Herpetic rash were classified as post-herpetic complications. Among the post-herpetic issues encountered in our study, the most prevalent was post-herpetic neuralgia (pain persisting for more than four weeks), observed in 31 (77.5%) patients, followed by post-herpetic pigmentation (macular) in 22 (55%) patients and scarring (papules, plaques, hypertrophic scars, and keloids) observed in 17 (42.5%) patients (Figure 6). Many patients reported both post-herpetic neuralgia and post-herpetic pigmentation or scarring (37.5%). Two patients exhibited the development of eczematous changes over the post-herpetic scar (Figure 4 and Figure 6). Upon presentation, the maximum duration of persistence of post-herpetic pigmentation and post-herpetic neuralgia observed in our study was seven years and three years, respectively. The most common type of post-herpetic neuralgia reported was the stabbing type in 13 (41.9%) patients, followed by the burning type in seven patients (22.5%), and the intensity was most frequently described as moderate in 18 (58%) patients (Figure 3). Sleep disturbances and hampering of day-to-day activities were reported by 14 (35%) patients. It was observed that the severity of the initial rash was directly proportional to the severity of pain and the duration of persistent symptoms. The early initiation of appropriate treatment resulted in a quicker resolution of symptoms.

**Figure 7:**
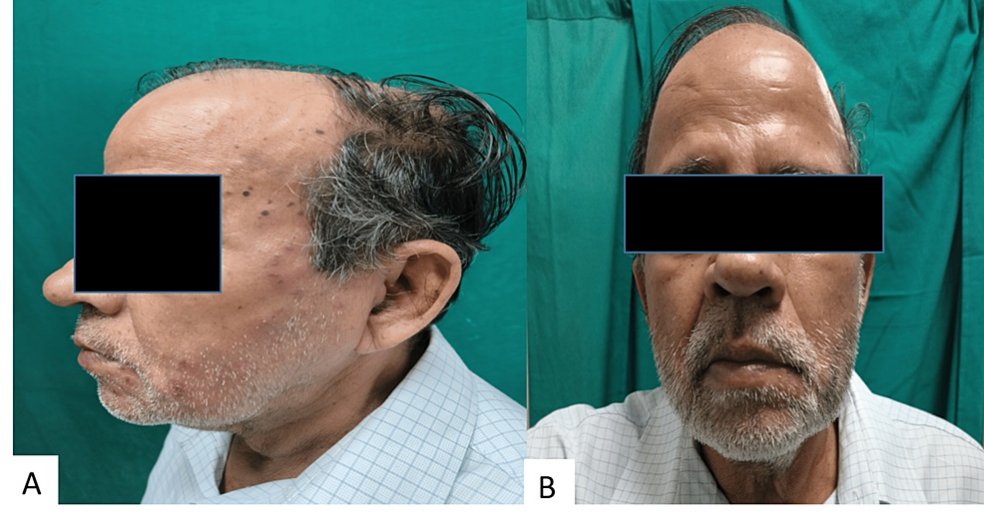
A and B: Clinical image of a patient showing facial asymmetry and grouped vesicles: Ramsay-Hunt syndrome

Ramsay-Hunt syndrome (also known as herpes zoster oticus (HZO)) is due to the re-activation of VZV infection in the geniculate ganglion of the facial nerve (Figure 7). It is characterized by the triad of ipsilateral facial paralysis, otalgia, and characteristic herpetic vesicles near the ear and the auditory canal [18].

**Figure 8:**
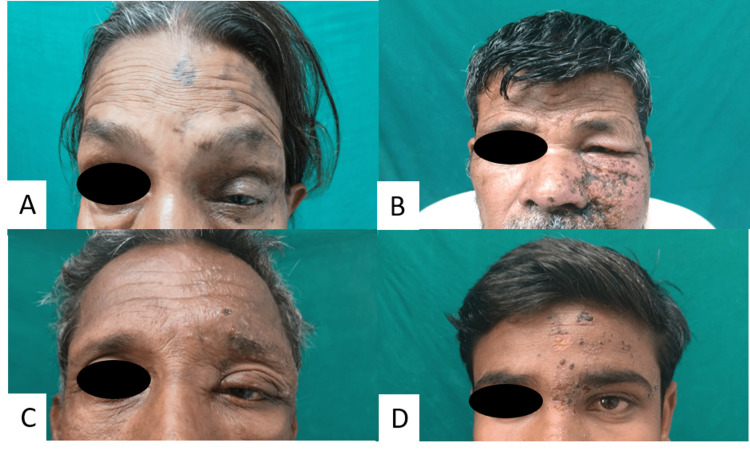
A, B, and C: Clinical image showing ophthalmological involvement in various patients due to herpes zoster (ptosis, congestion, watering)

Keratitis and conjunctivitis have been reported as common ocular manifestations of HZO in previous studies (Figure 8). Anterior uveitis has also been reported as a common intraocular manifestation of HZO [19,20,21]. The limitation of this study was that it is a single-center study.

While the diagnosis is primarily clinical and often involves Tzanck smear, in atypical presentations, real-time polymerase chain reaction (RT-PCR) for VZV in samples of vesicle fluid content, saliva, dry scabs, or even skin biopsy, as well as VZV direct staining with antibodies in infected cells or a smear of the base of a lesion, can also be performed to confirm the diagnosis [22].

Management of herpes zoster includes early intervention with antivirals such as acyclovir and valacyclovir, along with pain management [23]. Early diagnosis and initiation of antiviral therapy have been shown to reduce severity and complications, which was also observed in our study [24]. Pain management options for post-herpetic neuralgia (PHN) include anticonvulsants, antidepressants, topical therapies including lidocaine and capsaicin, and opioids, which are widely used in PHN treatment. Other measures like botulinum toxin, nerve blocks, spinal cord stimulation, and radiofrequency are also employed for PHN management [25]. Secondary prophylaxis with recombinant adjuvanted VZV glycoprotein E subunit vaccine or live attenuated VZV vaccine may also be considered as preventive options for this painful and debilitating disease [26].

## Conclusions

A broader understanding of the clinical and epidemiological factors of herpes zoster and post-herpetic complications is important, as this disease imposes a substantial burden on a tertiary care center across all age groups. Cases involving multi-dermatomal, severe, or unusual presentations, as well as widely disseminated lesions, should prompt an extensive work-up to rule out immune compromise. Common complications include post-herpetic neuralgia, unsightly scarring, and pigmentary changes. Untreated or sub-optimally treated cases are more susceptible to long-term aftereffects. Timely management of ophthalmological complications is crucial to prevent poor visual outcomes. Therefore, early diagnosis and initiation of appropriate antiviral therapy and pain management are key aspects of managing herpes zoster and post-herpetic neuralgia.
